# *Anopheles gambiae *genome reannotation through synthesis of *ab initio *and comparative gene prediction algorithms

**DOI:** 10.1186/gb-2006-7-3-r24

**Published:** 2006-03-27

**Authors:** Jun Li, Michelle M Riehle, Yan Zhang, Jiannong Xu, Frederick Oduol, Shawn M Gomez, Karin Eiglmeier, Beatrix M Ueberheide, Jeffrey Shabanowitz, Donald F Hunt, José MC Ribeiro, Kenneth D Vernick

**Affiliations:** 1 Center for Microbial and Plant Genomics, and Department of Microbiology, University of Minnesota, St Paul, MN 55108, USA; 2 Unité de Biochimie et Biologie Moléculaire des Insectes and CNRS FRE 2849, Institut Pasteur, 75724 Paris Cedex 15, France; 3 Department of Chemistry, McCormick Rd, University of Virginia, Charlottesville, VA 22904, USA; 4 Laboratory of Malaria and Vector Research, National Institute of Allergy and Infectious Diseases, Bethesda, MD 20892, USA

## Abstract

**Background:**

Complete genome annotation is a necessary tool as *Anopheles gambiae *researchers probe the biology of this potent malaria vector.

**Results:**

We reannotate the *A. gambiae *genome by synthesizing comparative and *ab initio *sets of predicted coding sequences (CDSs) into a single set using an exon-gene-union algorithm followed by an open-reading-frame-selection algorithm. The reannotation predicts 20,970 CDSs supported by at least two lines of evidence, and it lowers the proportion of CDSs lacking start and/or stop codons to only approximately 4%. The reannotated CDS set includes a set of 4,681 novel CDSs not represented in the Ensembl annotation but with EST support, and another set of 4,031 Ensembl-supported genes that undergo major structural and, therefore, probably functional changes in the reannotated set. The quality and accuracy of the reannotation was assessed by comparison with end sequences from 20,249 full-length cDNA clones, and evaluation of mass spectrometry peptide hit rates from an *A. gambiae *shotgun proteomic dataset confirms that the reannotated CDSs offer a high quality protein database for proteomics. We provide a functional proteomics annotation, ReAnoXcel, obtained by analysis of the new CDSs through the AnoXcel pipeline, which allows functional comparisons of the CDS sets within the same bioinformatic platform. CDS data are available for download.

**Conclusion:**

Comprehensive *A. gambiae *genome reannotation is achieved through a combination of comparative and *ab initio *gene prediction algorithms.

## Abstract 2

A comprehensive reannotation of the *Anopheles gambiae *genome using a combination of comparative and *ab initio *gene prediction algorithms has identified novel coding sequences.

## Background

Malaria, a mosquito-transmitted disease caused by parasites of the genus *Plasmodium*, infects as many as 500 million people per year. Approximately two million people die from malaria each year, with 75% of the deaths occurring in African children [1]. Human malaria parasites are transmitted by anopheline mosquitoes, of which *Anopheles gambiae *is the most prevalent vector in Africa. A thorough understanding of the *A. gambiae *genome and the genes and protein products integral to successful parasite transmission may inform malaria control strategies, including those capitalizing on natural malaria resistance and those using transgenic approaches.

There are two main approaches for gene prediction. Comparative algorithms such as Genewise [2] base gene prediction on similarity to known proteins, while *ab initio *prediction programs, such as GENSCAN [3], GeneMark [4] and SNAP [5], typically use the hidden Markov model (HMM) trained with known gene structures.

Comparative algorithms such as Genewise are inherently conservative because of their reliance on protein homology with other organisms and should yield predictions with higher specificity than non-comparative algorithms, but for the same reason their sensitivity is lower and they tend to underpredict the number of CDSs [2]. Comparative algorithms will particularly miss genes that display rapid evolutionary rates, including mosquito-specific genes that could control responses to mosquito-specific pathogens like malaria, or genes involved in human host-seeking or blood feeding. The paucity of CDS prediction in the current annotation has been noted by others [6,7]. In addition to under-prediction, comparative algorithms are known to have trouble predicting start/stop codons in flanking regions. This also results in a significant number of missing exons and split CDSs [2]. Conversely, *ab initio *gene prediction is quick, inexpensive, and not reliant on comparison with previously annotated genomes. The transcripts predicted by *ab initio *algorithms are normally complete and *ab initio *prediction results in at least partial prediction for about 95% of all genes, leaving fewer entirely missing genes [8]. On the other hand, due to the lack of comparison with known proteins, *ab initio *algorithms normally result in over-prediction, and unlike comparative algorithms, they do not provide information on alternative transcription.

The current Ensembl gene predictions for the *A. gambiae *genome sequence are an extremely important resource that has transformed malaria vector biology into a genomic discipline. The Ensembl predictions were generated as a consensus of automated pipeline results from Celera Otto [9] and Ensembl tools [10]. Both pipelines relied on the Genewise comparative algorithm and other comparative data sources for gene and protein prediction. Although the Otto pipeline employed some information from *ab initio *algorithms GRAIL, Genscan and FgenesH, this was only used "to refine the splicing pattern" of predicted genes [10] and the results of the *ab initio *programs "were not directly used in making the Otto predictions" [9]. Thus, Ensembl gene predictions for *A. gambiae *were directed by the comparative algorithm results, and can be considered a fundamentally comparative CDS set because *ab initio *algorithms did not add additional CDS content.

The Ensembl prediction pipeline was a reasonable and safe initial approach, because the comparative results would be expected to have high specificity for sequences that are expressed, if not high sensitivity for the complete number and extent of actual CDSs. However, the incomplete picture of genome annotation resulting from comparative prediction algorithms can make genomics and proteomics difficult, because ESTs, peptide catalogs or microarray features are not mapped to the correct genes or proteins. Inaccurate prediction of start and stop codons also raises issues for computational studies on gene regulatory sequence patterns because gene-flanking regions will be unknown.

Ideally, a conservative comparative approach could be used in a combinatorial manner with less conservative algorithms to yield the most comprehensive genome database without sacrificing accuracy. This report explores a combination of these two major gene prediction algorithms and provides reannotation of the *A. gambiae *genome through synthesis of *ab initio *and comparative gene prediction algorithms. This combinatorial approach yields a more complete CDS catalog, while retaining the high-specificity information content of the existing comparative prediction. The reannotation was evaluated for sensitivity, specificity, and biological information content using a large set of *A. gambiae *full-length cDNA sequences [7], RT-PCR, and a new proteomic dataset of mosquito mass spectrometry peptides.

## Results

### Synthesis of comparative and *ab initio *gene prediction algorithms

The GENSCAN, GeneMark and SNAP prediction tools utilizing *ab initio *algorithms yielded 32,020, 24,579, and 24,451 *A. gambiae *CDSs, respectively. The Ensembl database, based on the Genewise comparative algorithm, predicts 16,148 CDSs. To synthesize this set of 97,098 predicted CDSs into a single composite set, we used an exon-gene-union (EGU) algorithm and open-reading-frame-selection algorithm.

**Figure 1:**
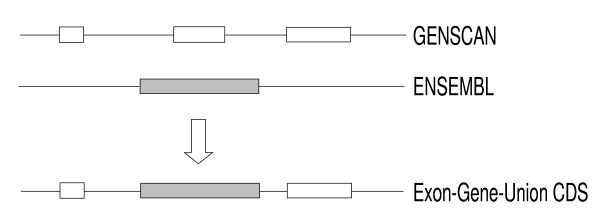
Diagram of EGU algorithm. The algorithm considers all exons predicted by GENSCAN and Ensembl as potential exons of a final CDS, and examines exon boundaries to assemble a new gene model. If exons from GENSCAN and Ensembl have different boundaries, the algorithm extends the exon boundary to include all nucleotides of the *ab initio *and comparative predictions. Subsequently, the ORF-selection algorithm (described in the text) chooses the best translatable reading frame to yield the final ReAnoCDS05 gene model.

First, CDSs predicted by GENSCAN and GeneWise were joined using the EGU algorithm (Figure 1). These two gene model sets were used because GENSCAN was found to be one of the most accurate *ab initio *gene prediction tools [11,12], and GeneWise was one of the most accurate comparative prediction methods [12]. The EGU algorithm can be summarized as: Base-pair of CDSs = base-pair predicted by Ensembl ∪ base-pair predicted by GENSCAN. The EGU algorithm involves two program steps: first, consider all the GENSCAN and Ensembl predicted exons as exons of a final CDS; and second, if exons from GENSCAN and Ensembl have different boundaries, extend the boundary to include all predicted base-pairs.

Because the newly predicted CDSs from the EGU algorithm do not necessarily have correct open reading frames (ORFs), an ORF-selection algorithm was used to select the best ORF according to the following criteria for ORF-selection implemented in three steps. In step 1, if more than 90% of a new CDS sequence can be translated directly without disruption by a stop codon, keep the transcript as the final CDS. In step 2, if the condition in step 1 is not met, select the predicted CDS from Ensembl, GENSCAN, GeneMark or SNAP that has the first initial exon and the last terminal exon and use this as the predicted CDS. In step 3, if neither steps 1 or 2 apply, select the predicted CDS from Ensembl, GENSCAN, GeneMark or SNAP that has the longest CDS and use this as the predicted CDS. These methods for synthesizing a number of predictions into a single re-annotation err on the side of inclusiveness by retaining the CDS with the greatest genomic extent between initial and terminal exons.

Through these combinatorial algorithms, we generated a total of 31,254 unique CDS predictions. Of these, 25,491 (81.5%) can be translated directly without interruption by internal stop codons, fulfilling step 1 of the ORF-selection algorithm above. About 11.5% (*n* = 3,583) have at least one ORF predicted from Ensembl, GENSCAN, GeneMark, or SNAP that covers the entire coding region despite possible differences in internal exons, fulfilling step (2) of the ORF-selection algorithm. Finally, the remaining 7% of predicted CDSs (*n* = 2,180) fulfilled step 3 of the ORF-selection algorithm, where the longest predicted CDS from Ensembl, GENSCAN, GeneMark or SNAP were selected to represent that CDS.

### ReAnoCDS05 reannotation dataset

Hereafter we refer to this new set of 31,254 CDSs as ReAnoCDS05 (Table 1). The ReAnoCDS05 dataset is freely available in the Artemis genome viewer [13] format and as FASTA format sequence databases (see Data availabilty in Materials and methods). In ReAnoCDS05, the average number of exons per gene is 4.98, greater than that of *Drosophila melanogaster *(4.65) and less than that of humans (10.14). Only 4% of predicted CDSs in ReAnoCDS05 lack start and/or stop codons, while in Ensembl 63% of CDSs are incomplete. Of the 31,254 CDSs predicted in ReAnoCDS05, 24,429 were located on chromosomes 2, 3 and X, and another 6,825 CDSs were located on the 'UNKN' virtual chromosome consisting of arbitrarily concatenated unplaced DNA contigs [10]. Some of the CDSs on the UNKN chromosome represent allelic forms of CDSs on known chromosomes [10,14], and others are probably contamination from bacterial symbionts [15].

### Detection of frame shifts in ReAnoCDS05

The 31,254 CDSs in ReAnoCDS05 initially included a small number of frame shifts relative to the original lines of evidence that were merged to generate the final prediction set. The frame shifts largely resulted from annotation errors in the original Ensembl predictions, for example, some introns comprised only one or two nucleotides, presumably to retain reading frame in the Ensembl gene models. The total rate of ReAnoCDS05 genes with frame shifts was about 0.6% (*n* = 190), which generated protein sequences slightly different from Ensembl or *ab initio *predictions prior to algorithm synthesis. Because the number of frame-shift cases was very small, however, they were corrected manually.

### Evaluation of ReAnoCDS05 by lines of supporting evidence

**Figure 2:**
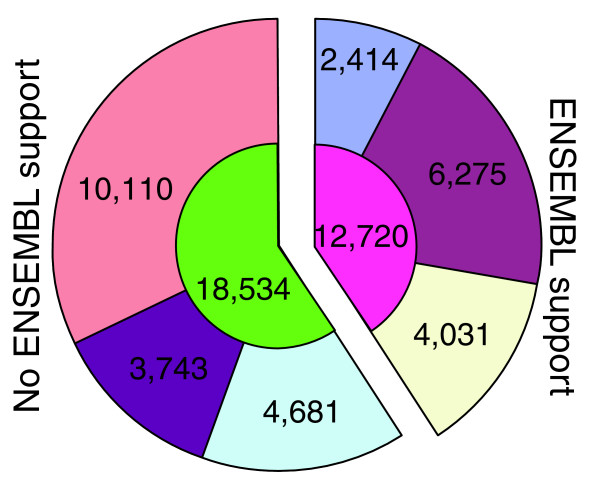
Comparison of ReAnoCDS05 and Ensembl CDS sets based on data sources. Total numbers of ReAnoCDS05 CDS predictions in each category related to data sources are indicated within pie slices. Inner ring 12,720, number of ReAnoCDS05 CDSs with Ensembl support; inner ring 18,534, ReAnoCDS05 CDSs without Ensembl support. Outer ring slices: 2,414, perfect match between ReAnoCDS05 and Ensembl predictions; 6,275, ReAnoCDS05 CDSs that extend and/or merge Ensembl CDSs; 4,031, ReAnoCDS05 CDSs that involve major structural changes or reorganization in the overlapping Ensembl CDS(s), where Ensembl CDSs undergo combinations of boundary change, internal exon loss/gain/change, and splitting to >1 ReAnoCDS05 CDS; 4,681, novel ReAnoCDS05 CDSs with NCBI dbEST support; 3,743, novel ReAnoCDS05 CDSs without EST support but with >1 line of *ab initio *support; 10,110, ReAnoCDS05 CDS with only 1 line of *ab initio *support.

All CDSs in ReAnoCDS05 were classified based on both empirical and *in silico *lines of supporting evidence (Figure 2). In addition to those CDSs with Ensembl support (*n* = 12,720), there are 4,681 novel CDSs with EST support and 3,743 novel CDSs predicted by at least two *ab initio *algorithms. The latter set of 3,743 CDSs is based upon GENSCAN predictions, and is supported by predictions of one or both of the other *ab initio *algorithms used. Of predicted ReAnoCDS05 CDSs, 67% (*n* = 20,970) have more than one line of supporting evidence while 33% (*n* = 10,284) have only one line of supporting evidence. Of these latter single-evidence predictions, 174 are supported only by Ensembl, and the remaining 10,110 are *ab initio *predictions supported only by GENSCAN. Of the 10,284 single-evidence CDSs, 28% are assigned to the UNKN chromosome.

We subdivided ReAnoCDS05 into two subsets based on lines of supporting evidence: the High-Quality (HQ-CDS) dataset of CDSs with ≥2 lines of support (*n* = 20,970), and the Low-Quality (LQ-CDS) dataset of CDSs with only one line of support (*n* = 10,284). The relative biological information content of these prediction sets is functionally evaluated by proteomic assay below.

### Validation of ReAnoCDS05 predictions by full-length cDNA dataset

**Figure 3:**
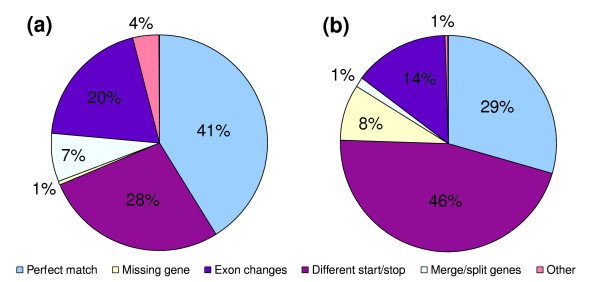
Manual comparison of ReAnoCDS05 and Ensembl based on a set of full-length cDNA sequences. The charts show the analysis of all cDNAs in the dataset mapped to the X-chromosome (*n* = 800), corresponding to 156 cDNA loci, and their conceptual translation products in relation to CDSs predicted by **(a) **ReAnoCDS05 and **(b) **Ensembl. Categories of comparison indicated in the legend are: perfect match, proportion of cDNA sequences with translation products that display exact match to predicted peptide sequence of annotation CDS; missing gene, cDNAs not represented by a corresponding annotation CDS; exon changes, cDNAs for which the corresponding annotation CDSs display extra exons, missing exons, and/or exon boundary changes; different start/stop, cDNA loci for which annotation CDSs display different predicted translation initiation and/or termination; merge/split genes, cDNA loci that overlap multiple annotation CDSs, or vice versa; other, including multiple low-frequency cases.

A set of 20,249 full-length cDNA sequences generated as paired contigs [7] were used as a validation test for accuracy of the ReAnoCDS05 reannotation. The 20,249 paired contigs were mapped to 1,885 ReAnoCDS05 CDSs and 2,257 Ensembl CDSs. The number of genes mapped by the paired contigs is smaller than the total number of query sequences because many genes were hit by paired contigs multiple times. Automated comparison of the nucleotide sequences of mapped cDNAs and the ReAnoCDS05 and Ensembl CDSs indicated that 1% of cDNA transcripts placed on the Golden Path sequence were missing from ReAnoCDS05, while 5% were missing from Ensembl, and 45% of ReAnoCDS05 CDSs were annotated completely correctly (exact match of all exon boundaries including start/stop codons), while 30% of Ensembl CDSs met this criterion (Table 1). To extend this analysis, the cDNAs (*n* = 800) mapped to the X chromosome (*n* = 156 loci) were used in a detailed manual examination of ReAnoCDS05 and Ensembl CDS support by the cDNA nucleotide sequences and their conceptual translations (Figure 3). Results of the manual analysis were consistent with the automated results, again showing a greater level of precise exon structural and sequence match between cDNAs and ReAnoCDS05 (41%) compared to Ensembl (29%). In this manual analysis, the overall sensitivity of ReAnoCDS05 is 0.99 and of Ensembl 0.92. The manual analysis also indicated that the increased perfect-match level of ReAnoCDS05 was largely due to greater accuracy of start/stop codon prediction by ReAnoCDS05 (28% ReAnoCDS05 and 46% Ensembl disagreement, respectively, with the translated X-chromosome cDNA dataset).

The overall specificity of the Ensembl CDS predictions for *A. gambiae *has not yet been reported. It is difficult to accurately estimate the specificity of either CDS dataset, ReAnoCDS05 or Ensembl, because the *A. gambiae *genome does not have any exhaustively characterized model regions, analogous to the 30 Mb ENCODE [16] and 2.9 Mb GASP [17] projects in human and *Drosophila*, respectively, that could serve as a benchmark denominator for determination of specificity. For the purpose of comparison, however, here we assign to the Ensembl CDSs an overall nucleotide specificity of 0.99, which was derived from a test of GeneWise detection of experimental CDSs embedded in semi-artificial genomic sequences [18]. Then, we devised a method to estimate ReAnoCDS05 nucleotide specificity by using the amount of supporting evidence to separate true positive from false positive CDSs, assuming that the majority of single-evidence CDSs in LQ-CDS are false positive (see Materials and methods). The resulting nucleotide specificity for ReAnoCDS05 is calculated to be 0.96, compared to 0.99 for Ensembl (Table 1).

### Validation of ReAnoCDS05 predictions by RT-PCR

**Figure 4:**
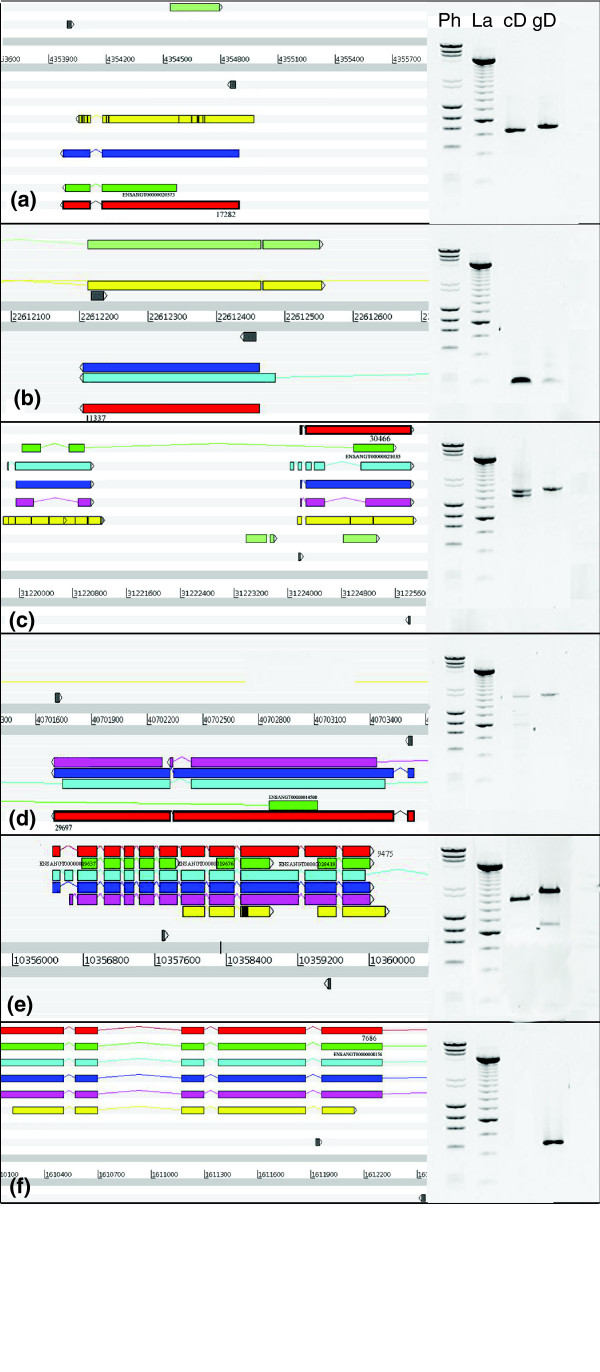
Validation of ReAnoCDS05 predictions by RT-PCR. Differences between ReAnoCDS05 and Ensembl CDS predictions were experimentally tested by RT-PCR using *A. gambiae *cDNA or gDNA as templates. The left side of each panel is a map of CDS predictions and supporting lines of evidence, and the right side is a reverse-color image of, from left to right lanes, PhiX/Lambda DNA size standard (Ph), 250 bp DNA ladder (La), and PCR performed on either cDNA (cD), or gDNA template (gD). **(a-e) **Five cases of potential annotation difference were tested (described in Results); **(f) **control to test for gDNA contamination of cDNA using primers in two predicted introns to amplify across the intervening exon. In each case except the control, the ReAnoCDS05 and Ensembl annotations made different predictions for the RT-PCR result using cDNA template (in all cases gDNA was the positive control), as follows: (a) ReAnoCDS05 predicted 815 bp, Ensembl predicted no product, RT-PCR estimated 815 bp; (b) ReAnoCDS05 predicted 241 bp, Ensembl predicted no product, RT-PCR estimated 241 bp; (c) ReAnoCDS05 predicted 1,555 bp, Ensembl predicted no product, RT-PCR estimated 1,555 bp; (d) ReAnoCDS05 predicted 1,822 bp, Ensembl predicted no product, RT-PCR estimated 1,822 bp; (e) ReAnoCDS05 predicted 1,600 bp, Ensembl predicted no product, RT-PCR estimated 1,600; (f) both ReAnoCDS05 and Ensembl predicted no product, and no product was present. Left panel key: red bars, CDSs from ReAnoCDS05 re-annotation (numbers are ReAnoCDS05 unique IDs); dark green bars, CDSs from Ensembl (with ENSANGT transcript IDs); dark blue bars, CDS from GENSCAN; light blue bars, CDSs from GeneMark; pink bars, CDSs from SNAP; yellow bars, dbEST contigs; light green bars, ESTs from immune-enriched cDNA library [45]. All bars on map depict CDSs only, except EST and SNAP, which may also contain UTR sequences. Small gray arrowheads indicate the locations of primers used for verification of CDS structure. Ensembl nucleotide coordinates are shown for the indicated chromosomes.

RT-PCR was used as an additional empirical validation method for a small set of genes to verify ReAnoCDS05 CDSs and evaluate differences with the current Ensembl annotation (Figure 4). RT-PCR assays were designed at sites where ReAnoCDS05 and Ensembl predict different CDS structures, so that the presence and size of product bands unambiguously verify one of the CDS predictions. Maps of the ReAnoCDS05 and Ensembl CDS predictions for the five test categories are shown in Figure 4 (left side of each panel). Five categories of potential difference between ReAnoCDS05 and Ensembl were tested, as follows (corresponding to Figure 4a-e): altered 5' and/or 3' boundaries of ReAnoCDS05 CDSs introduce potential start and/or stop codons not present in Ensembl (Figure 4a); novel ReAnoCDS05 CDSs without Ensembl support (Figure 4b); Ensembl CDSs split into >1 ReAnoCDS05 CDSs (Figure 4c); major structural changes or reorganization in an Ensembl CDSs yields ReAnoCDS05 CDSs with major differences from Ensembl (Figure 4d); >1 Ensembl CDSs merged into 1 ReAnoCDS05 CDS (Figure 4e). Each assay included a positive control reaction with genomic DNA (gDNA) template. A negative control assay in which ReAnoCDS05 and Ensembl both predict no product verified the absence of gDNA contamination in the cDNA template (Figure 4f). In the cases tested, RT-PCR products confirmed the ReAnoCDS05 CDS predictions as compared to the alternative Ensembl predictions. This experimental result complements the validation provided by automated and manual analyses using the larger full-length cDNA dataset above. Although anecdotal rather than quantitative, the RT-PCR analysis at least indicates that these five types of annotation changes actually exist as predicted by ReAnoCDS05.

### ReAnoCDS05 improves *A. gambiae *proteomic coverage

We generated 8,103 high quality *A. gambiae *hemolymph peptide sequences by tandem mass spectrometry (MS/MS). Of these peptides, 62% (5,020) do not map to Ensembl proteins, compared to 12% (873) that do not map to ReAnoCDS05. Thus, a dataset of MS/MS peptides was more efficiently populated with cognate protein identities from ReAnoCDS05 than Ensembl, and, therefore, ReAnoCDS05 significantly improved *A. gambiae *genome annotation coverage.

To determine the basis of the apparently greater information content of ReAnoCDS05 in the MS/MS experiment, we compared the biological information content of the two ReAnoCDS05 CDS subsets (multiple-evidence HQ-CDS and single-evidence LQ-CDS) with Ensembl CDSs using a peptide hit index (PHI; see Materials and methods) to determine the MS/MS peptide hit rates in each database. The PHI of the HQ-CDS database (0.305) was greater than that of the Ensembl database (0.190), while the LQ-CDS database displayed the lowest value (0.079). The LQ-CDS dataset should contain a relatively small proportion of correct CDS predictions because the dataset is based on a single line of *ab initio *support [19]. The low PHI score of the LQ-CDS dataset is consistent with this expectation. Moreover, when PHI scores are normalized to numbers of amino acid residues in each database, the relative rank of each database remained the same (values for (peptide hits/total amino acids in database) × 1,000 are 0.54 for HQ-CDS, 0.45 for Ensembl, and 0.28 for LQ-CDS). This result indicates that the higher PHI score for HQ-CDS is not a consequence of the longer mean CDS length in ReAnoCDS05 compared to Ensembl. This analysis partitions ReAnoCDS05 into high- and low-quality components in terms of biological information content, and indicates that the HQ-CDS CDS dataset specifically enriches the biological information that can be extracted from MS/MS proteomic data as compared to the Ensembl dataset.

### ReAnoCDS05 and protein functional annotation

To facilitate data mining and functional annotation of the proteome set, all predicted ReAnoCDS05 proteins were organized in a hyperlinked Excel spreadsheet database, named ReAnoXcel. ReAnoXcel is available for download (see Materials and methods). The ReAnoXcel database contains numerous categories of information for each CDS translation product, including presence or absence of signal peptides indicative of secretion [20], transmembrane domains [21], molecular weight, pI, genome location, and various comparisons to other protein and motif collections, such as the NCBI non-redundant protein database, Gene Ontology [22], CDD [23], and homology to proteins of other organisms, including bacteria, as done before in AnoXcel for the Ensembl proteome set [24].

The ReAnoCDS05 proteome was also compared to the set of 162,565 *A. gambiae *EST sequences from dbEST (NCBI) and TIGR and assembled into 34,107 contigs and singletons using a combination of the tools BLASTN [25] and the CAP3 assembler [26] as indicated before [27], facilitating verification of the proteome data set. Additionally, the number of sequences from each EST library mapping to unique proteins is indicated. For example, the spreadsheet column named 'Head-all' (including several libraries made from the head of adult mosquitoes) can be sorted to find those proteins with high expression in the adult mosquito head, or the column named 'Blood-fed' (representing approximately 40,000 ESTs of 24 hours post-blood fed mosquitoes) can be compared to the column named 'Non blood-fed' (similar number of ESTs deriving from sugar-fed adult mosquitoes) to find those proteins more expressed after the bloodmeal [28,29]. A microarray experiment using the Affymetrix whole-genome chip [30] is also mapped to the dataset.

Here we provide only a few possibilities of how ReAnoXcel can be used in data mining. For example, comparison of the reannotated ReAnoCDS05 proteome with the Ensembl set using BLASTP without the low complexity filter identified 1,312 ReAnoCDS05 proteins where the corresponding Ensembl proteins displayed 100% sequence identity but only 50% to 99% of the length of the ReAnoCDS05 proteins. Within these latter 1,312 proteins, apparently truncated in Ensembl, the number of ReAnoCDS05 protein sequences with predicted signal peptides indicative of secretion was 281 in comparison with 211 in the Ensembl set, suggesting that the additional extent of the ReAnoCDS05 proteins is biologically meaningful. Also within the 1,312 set, the average number of membrane helices as predicted by the program TMHMM [21], excluding 0 and 1 helices from both sets, was 5.4 ± 0.29 and 3.7 ± 0.23 (mean ± standard error, *n* = 214) for ReAnoCDS05 and Ensembl, respectively. In particular, 13 proteins in the ReAnoCDS05 set appeared with 7 transmembrane (7TM) domains, none of which were predicted to be 7TM in the Ensembl set. This is relevant because many proteins containing 7TM domains are membrane receptors [31]. Indeed, the totality of the ReAnoCDS05 set has 159 proteins with predicted 7TM domains, only 86 of which are also predicted as 7TM in the Ensembl set.

Comparison of the proteomes of *A. gambiae *and *D. melanogaster *indicated, among other differences, a mosquito expansion of proteases of the trypsin family [32]. These enzymes are involved in protein digestion in the midgut and also in signal transduction and the regulation of proteolytic cascades leading to tissue development and immunity. Digestive trypsins are usually small (approximately 200 to 250 amino acids), while regulatory proteases have additional domains leading to larger proteins. Comparison of the Ensembl proteome set with ReAnoCDS05 shows 318 proteins with the PFAM signature in the Ensembl set, compared with 311 from the ReAnoCDS05 set. In the Ensembl set, 31 proteins overlap with others in their chromosome locations, indicating different predictions of the same gene region, while the ReAnoCDS05 set has 43 such overlapping gene products. Although the two sets have a similar number of predicted trypsins, the Ensembl set has 12 proteins that do not produce identical predictions in ReAnoCDS05, and ReAnoCDS05 produces 65 proteins not predicted in the Ensembl set. Additionally, the average size of the trypsins in the Ensembl set is 298 amino acid residues, while the ReAnoCDS05 set has an average size twice as large, with 687 residues, indicating the possibility that the ReAnoCDS05 set identifies more larger, regulatory trypsins. These comparisons indicate that the ReAnoCDS05 set extends the predictions of the trypsin family in *A. gambiae*, potentially with better detection of larger regulatory enzymes.

The ReAnoXcel spreadsheet may also facilitate discovery of transposable elements and bacterial transcripts compared to the Ensembl set. Searching for transposons (by searching the strings 'rve,' 'RTV," and 'transposase_' on the CDD results) retrieves 2,896 sequences in the ReAnoCDS05 set as opposed to 132 in the Ensembl database. Also, because the shotgun approach to sequencing the *A. gambiae *genome used DNA from adult mosquitoes colonized with bacteria, there are many DNA sequences derived from these bacterial symbiont genomes. Recently, whole symbiont genomes were retrieved from shotgun sequencing of *Drosophila *genomes [15]. To help retrieve these sequences of bacteria associated with *A. gambiae*, the spreadsheet can be sorted on the best value to NCBI bacterial proteomes, thus yielding 4,655 proteins with BLASTP E-values of 1E-15 or lower. Sorting this subset on the 'chromosome' column retrieves 1,240 sequences on 'UNKN' and further sorting on the taxonomic column facilitates removal of non-bacterial matches to obtain a set of 952 mostly likely bacterial proteins. Resorting of this dataset on the gene 'start' column allows identification of segments of bacterial genomes mapped to the UNKN chromosome, which carries >86% of the high-scoring bacterial homologs.

## Discussion

Researchers attempting to dissect the biology of anopheline mosquitoes, particularly their role in malaria parasite transmission, rely heavily on the Ensembl *A. gambiae *gene annotation. The current Ensembl annotation, while an extremely valuable tool, is prone to incomplete CDS prediction and missing CDSs due to the use of comparative algorithms in CDS annotation. This results in difficulties for genomics, genetics and proteomics.

Comparative gene prediction algorithms yield annotations with high specificity and reliability, while *ab initio *gene prediction algorithms provide more comprehensive and sensitive but less specific annotations [8]. In an attempt to generate more complete *A. gambiae *genomic information, we synthesized results from these two major classes of algorithms to create a single set of re-annotated CDSs, called ReAnoCDS05. This combinatorial algorithm balances reliable CDS prediction resulting from comparative algorithms with comprehensive CDS prediction from *ab initio *algorithms. Synthesizing results from the two major algorithm types may complement the weaknesses of either approach used in isolation. For example, Otto predicted gene boundaries on the basis of "overlapping protein and EST matches" [10] while ReAnoCDS05 predicted gene boundaries by EGU. The ReAnoXcel database presented here facilitates comparative analysis of ReAnoCDS05/ReAnoXcel and Ensembl/AnoXcel datasets within the same bioinformatic platform.

We used automated and manual curation of full-length cDNAs to estimate the sensitivity of ReAnoCDS05 and Ensembl. Empirical validation with these datasets showed that the accuracy of predicted CDSs in ReAnoCDS05 was improved (from 30% to 45%), and overall sensitivity was also improved (from 0.92 to 0.99). However, it should also be noted that the Ensembl annotation is three years old and a larger number of *A. gambiae *EST sequences are now available for the ReAnoCDS05 annotation than for the original Ensembl project. This is undoubtedly a factor in the higher sensitivity of ReAnoCDS05 as compared to Ensembl predictions. The synthesis algorithm resulted in thousands of new CDSs with other empirical or computational support, and, therefore, it increases genome annotation coverage. Manual RT-PCR on a small sample set of CDSs indicates that all tested classes of CDS changes in ReAnoCDS05 as compared to Ensembl actually exist.

We functionally tested the utility of the ReAnoCDS05 CDS dataset for MS/MS peptide analysis. In this analysis, we divided ReAnoCDS05 into two subsets based on amount of supporting evidence. Most of the ReAnoCDS05 biological information content is concentrated in the ReAnoCDS05 HQ-CDS subset with ≥2 lines of support, containing 20,970 predicted CDSs, which permitted extraction of more information from a set of MS/MS spectra than did searching the Ensembl or single-evidence ReAnoCDS05 LQ-CDS CDS databases. We consider the 20,970 CDSs in the ReAnoCDS05 HQ-CDS dataset to be the most informative and balanced current version of the *A. gambiae *CDS set.

It is difficult to estimate specificity of gene prediction in a less mature genome annotation like *A. gambiae*, which lacks well-annotated reference genome regions. Alternatively, specificity could be indirectly estimated using a computationally constructed semi-artificial genome sequence, in which known 'CDSs' are interspersed in synthetic 'intergenic sequences' [18]. In this context, locations of 'actual CDSs' are known and both specificity and sensitivity of different prediction pipelines can be compared, although subject to limitations based on the highly artificial test system. The specificity value we assign to the Ensembl CDSs was from GeneWise prediction of CDSs from such an artificial set. However, a proper comparison with ReAnoCDS05 by this approach is problematic because key components of the Ensembl prediction pipeline (for example, Otto) are proprietary. Consequently, we devised a way to obtain an estimate of ReAnoCDS05 specificity by using amount of supporting evidence to distinguish true positive and false positive CDSs, and the specificity of ReAnoCDS05 at the nucleotide level is calculated to be 0.96, which is lower than specificity of Ensembl (0.99). These values are approximate, but are consistent with the expectation that Ensembl CDSs, based as they are on comparative annotation, should have high specificity, and that ReAnoCDS05 CDS may be overpredicted.

Unlike a previously reported approach to combine two *ab initio *algorithms [11], our combination of both comparative and *ab initio *algorithms aimed to preserve as much information as possible from both algorithms, and we required that ReAnoCDS05 gene models did not discard any information from the Ensembl data source. This requirement may lead to distortions in predictions for some genes, which could be repaired based on new empirical data (for example, EST or MS/MS). It is also expected that using the GENSCAN algorithm trained on *A. gambiae *data would improve prediction accuracy, because GENSCAN as utilized is trained on human data.

The different annotations have distinct features, and researchers need to decide which CDS information to use based on the application at hand. In particular, the 10,110 predicted ReAnoCDS05 CDSs supported by only one line of *ab initio *evidence are likely to have a relatively high rate of overprediction. This is confirmed by the low EST and MS/MS peptide hit rates to the ReAnoCDS05 LQ-CDS protein dataset, and is also consistent with the outcome of similar classes of predictions in other systems [19]. We do not recommend routine use of LQ-CDS except for applications that forgive overprediction (for example, bioinformatic homology searches). On the other hand, the high rate of MS/MS peptide information in the 20,970 CDSs of ReAnoCDS05 HQ-CDS compared to Ensembl clearly indicates that HQ-CDS is the preferred existing protein database for *A. gambiae *proteomics.

## Conclusion

Overall, the synthesis algorithm implemented to produce the current reannotation may be useful in directing the annotation of other new genomes, and the reannotated *A. gambiae *CDSs presented in this paper will provide a useful resource, complementary to the Ensembl database, for mosquito biology.

## Materials and methods

### *A. gambiae *CDS and EST data preparation

The *A. gambiae *Golden Path sequence and annotation was downloaded from Ensembl (database release version 26.2b.1, November 2004, based on sequence assembly MOZ2a) [33]. Golden Path sequence and nucleotide coordinates remain identical in Ensembl database release 35.2 g, the current version at the time of manuscript revision (November 2005). The gene prediction tool GENSCAN with an HMM trained by human genes was used to predict CDSs in the *A. gambiae *Golden Path sequence. The Golden Path was also analyzed using GeneMark.hmm (GeneProbe Inc., Atlanta, GA, USA) using an HMM trained by *Drosophila melanogaster *genes. The exons predicted by Ensembl/GeneWise and CDSs predicted by SNAP (an algorithm trained by selected *A. gambiae *EST genes [34]) were obtained from Ensembl [33]. The complete dbEST database of *A. gambiae *ESTs (*n* = 134,784, January 2005) was downloaded from NCBI [35], and ESTs were clustered and contigged into 11,697 contigs (≥2 ESTs) and 15,645 singlets using PaCE [36] and CAP3 [26]. The SNAP CDSs, EST contigs and EST singlets were mapped onto the *A. gambiae *Golden Path using BLAT [37]. The reannotation set of 31,254 CDSs were scored by Ensembl, GENSCAN, GeneMark, SNAP, and a comprehensive set of *A. gambiae *ESTs to give each CDS a reliability score. All the sequence and mapping information was stored in a MySQL database [38]. Functional proteomic analysis was carried out using the previously described AnoXcel pipeline [24].

### Full-length cDNA assembly and mapping

Paired end-sequences generated from the 3'-end and 5'-ends of 31,424 full-length cDNA clones were generated by an Institut Pasteur/Genoscope project [7]. The sequences can be obtained from the NCBI nucleotide database by a search with the key words 'HTC [Keyword] AND Genoscope [Author] AND Anopheles gambiae [Organism] AND extremity [Text Word] and full-length [Text Word]'. The end sequences for a given clone, which were single-pass sequences from each end of a full-length cDNA clone, were paired based on the clone name. If the end-pairs overlapped by at least 35 base-pairs (bp) they were contigged, and all such paired contigs that were mapped to a chromosome at ≥90% nucleotide identity constituted a dataset of 20,249 presumptively full-length cDNA sequences that were used to verify the re-annotation (duplicated sequences from the same gene were not collapsed). Only paired contigs (rather than non-overlapping cDNA ends) were used in this analysis because they contained the external boundaries and internal exon structure of the cognate CDS.

### Estimation of sensitivity and specificity

The precise and overall sensitivity of ReAnoCDS05 and Ensembl CDS sets were obtained by manual curation and examination of all full-length cDNAs (*n* = 800) that mapped to the X chromosome (*n* = 156 loci). Precise sensitivity is the proportion of overlapped CDS precisely matching cDNA pair contig boundaries. Overall sensitivity is the proportion of cDNA pair contigs displaying any overlap to a CDS.

For specificity, we devised a method to provide an estimate of nucleotide specificity of ReAnoCDS05 by using the amount of supporting evidence to separate the true positives from false positives in ReAnoCDS05. For this purpose, we assumed that the multiple-evidence HQ-CDS dataset (41,772,749 bp of predicted CDS length) were true positive, that the single-evidence LQ-CDS dataset (9,414,555 bp of predicted CDS) were false positive, that 417,727 bp were false negative based on overall sensitivity of 0.99, and that the remaining 240,981,817 nucleotides of the genome sequence, which lack predicted CDSs, are true negative actually devoid of CDSs (from total genome length including UNKN 292,586,848 bp minus HQ-CDS 41,772,749, LQ-CDS 9,414,555, and 417,727 false negative). Thus, the nucleotide length of LQ-CDS is regarded as the false positive subset within ReAnoCDS05, which although not exactly correct, is probably reasonably correct. Then, specificity is estimated as 240,981,817 CDS-devoid nucleotides divided by (240,981,817 CDS-devoid plus 9,414,555 false-positive nucleotides) = 0.962. For Ensembl nucleotide specificity, we accepted the value, 0.99, reported for GeneWise prediction on semi-artificial genomic sequences [18].

### RT-PCR verification

Total RNA was isolated from a pool of female mosquitoes using Trizol Reagent (Invitrogen, Carlsbad, CA, USA) and mRNA was purified using Oligotex (Qiagen, Valencia, CA, USA). The pool of female mosquitoes included sugar-fed mosquitoes, mosquitoes fed on normal or malaria-infected bloodmeals, mosquitoes injected with bacterial elicitor lipopolysaccharide, and mosquitoes injected with saline. This pool of mosquitoes was used to enrich the representation of transcripts expressed under diverse conditions. All mRNA was treated with RNase-free DNase (Invitrogen) to remove contaminating genomic DNA and the DNase was heat inactivated prior to cDNA synthesis. cDNA synthesis was performed using Superscript III Reverse Transcriptase (Invitrogen) and resulting cDNA was used as template in PCR reactions. Primer pairs were designed using Primer3 [39] and spanned an intron where possible. PCR was performed with Accuprime II polymerase (Invitrogen), 25 to 50 ng cDNA template and 300 nM of each primer using the following cycle: 95°C for 2 minutes; 35 cycles of 94°C for 45 seconds, 55°C for 45 seconds and 68°C for 3 minutes. Products were analyzed by electrophoresis to determine presence or absence of product fragment, and fragment lengths were estimated in relation to two DNA size standards, PhiX-HaeIII+Lambda-HindIII, and 250 bp ladder (Invitrogen). Primer sequences used (numbered 'forward' and 'reverse' according to Figure 4): 5a-for, AATAAAAGTTGCAGTTATCTGTGCT; 5a-rev, ACGGCCGTATCATCATTTTG; 5b-for, CATGCTGTTGGCCGTGTC; 5b-rev, CACGGTGGCCACAATGAT; 5c-for, GTGGTGTGCACTCCTCAAGA; 5c-rev, ATTCCGCGTTCGCACACT; 5d-for, TTACGCGCCGTATCACAAAT; 5d-rev, GTCTGTGATTGCCGAGCTG; 5e-for, AGATGAAGCTGCTTGCCAAT; 5e-rev, ATTGCCGTTGGTACGATCTC; 5f-for, AAACGTTTTGTTTGCGGTTT; 5f-rev, TCTCGCTCACACAAACATGC.

### Mass spectrometry and peptide analysis

For MS/MS, mosquito tissue extracts were treated with trypsin, fractionated by HPLC and analyzed using an LCQ quadrupole ion trap mass spectrometer (Thermo-Finnigan, San Jose, CA, USA). Details and full results will be presented elsewhere. MS/MS spectra were first searched using SEQUEST [40] against all *A. gambiae *predictions from three *ab initio *models (GENSCAN, GeneMark, SNAP) and Ensembl combined as a single protein database to yield a dataset of peptide sequences corresponding to MS/MS spectra. Next, the post-SEQUEST peptide dataset (n = 34,438) was filtered according to the criteria (Xcorr > 1.5 and charge ≥ 2) or (Xcorr > 2.0 and charge = 1) to yield a high-quality peptide dataset (n = 8103). This peptide dataset was used to assay protein database quality by searching protein databases for perfect sequence match adjacent to a predicted trypsin cleavage site. We used a PHI to quantify the biological information content of protein databases for MS/MS peptide data. The PHI was defined as the total number of MS/MS peptide matches to proteins in a database, divided by the total number of proteins in the database.

### Data availability

The ReAnoCDS05 reannotation and contributing lines of evidence are available for download from [41,42]. The data are posted as General Feature Format (GFF) files that can be opened, viewed and edited in the Artemis genome viewer [13], (available at [43]). FASTA files of sequence databases are also posted for download. The sequence databases are nucleotide and protein for CDSs in the HQ-CDS dataset with at least two lines of support (*n* = 20,970), CDSs in the LQ-CDS dataset with one line of support (*n* = 10,284), or the entire ReAnoCDS05 CDS dataset (*n* = 31,254). In addition, the ReAnoXcel protein functional annotation spreadsheet can be downloaded from NCBI [44].

Regarding the GFF files, note that Artemis for Windows is set by default to use a maximum of 150 Mb of memory, which is not sufficient to open the ReAnoCDS05 files. The largest ReAnoCDS05 file, chromosome 2R, requires about 1 Gb of memory. The solution is to create a shortcut to Artemis, and edit the properties of the shortcut to add "java -mx1000 m -jar" (without the quotation marks) to the beginning of the Target: field. Thus, the Target: field will appear as Target: java -mx1000 m -jar "C:\Program Files\Artemis\artemis_v7.jar". Run Artemis from the shortcut (assuming the machine has >1 Gb of memory). A similar issue exists when running Artemis under Linux/UNIX, where the art script needs to be edited from MEM = "-mx150 m -ms20 m" to MEM = "-mx1000 m -ms20 m". See FAQ in Artemis online documentation.

ReAnoXcel can be opened in Microsoft Excel and most fields are hyperlinked to original data sources. ReAnoXcel structure is based on AnoXcel [24] and the same general instructions for use apply.

## Figures and Tables

**Table 1:** Comparison of ReAnoCDS05 and Ensembl

	ReAnoCDS05	Ensembl
Total CDSs	31,254	16,148
Total exons	155,680	58,579
Average exons per CDS	4.98	3.62
CDS completion rate*	96%	37%
CDSs overlapped by cDNA pair contigs^†^	1,885	2,257
CDSs perfectly matched to cDNA pair contigs^†^	752	672
cDNA pair contigs not overlapping any CDSs^†^	1%	8%
Specificity (nucleotide)^‡^	0.96	0.99
Sensitivity (overall)^§^	99%	92%
Sensitivity (perfect)^¶^	45%	30%
Protein hits yielded by MS peptides	4,737	1,413
MS peptide missing rate^¥^	12%	62%

*Proportion of CDSs with start and stop codon. ^†^Paired end sequences of full-length cDNAs from [7]. ^‡^Calculation for ReAnoCDS05 described in Materials and methods, Ensembl value from [18]. ^§^Proportion of cDNA pair contigs overlapping a CDS. ^¶^Proportion of overlapped CDSs precisely matching cDNA pair contig boundaries. ^¥^Proportion of mass spectrometry (MS) peptides failing to hit any protein in database.
